# Trazodone modulates behavioral alterations in scopolamine-induced cognitive deficit by targeting brain-derived neurotropic factor and cAMP response element-binding protein signaling

**DOI:** 10.3389/fncel.2025.1681080

**Published:** 2026-01-02

**Authors:** Prashant Dhaka, Pinky, Neha, Mohammad Ahmad Khan, Syed Arman Rabbani, Mohamed El-Tanani, Suhel Parvez

**Affiliations:** 1 Department of Toxicology, School of Chemical and Life Sciences, Jamia Hamdard, New Delhi, India; 2 Department of Pharmacology, School of Pharmaceutical Education and Research, Jamia Hamdard, New Delhi, India; 3 Department of Clinical Pharmacy and Pharmacology, RAK College of Pharmacy, RAK Medical and Health Sciences University, Ras Al Khaimah, United Arab Emirates; 4 RAK College of Pharmacy, RAK Medical and Health Sciences University, Ras Al Khaimah, United Arab Emirates

**Keywords:** trazodone, scopolamine, neurodegeneration, neuroprotection, Alzheimer’s disease

## Abstract

**Background:**

Trazodone, an antidepressant, may play a potential role in enhancing long-term memory by combining anxious behavior deficits induced by scopolamine. The current study proposes the potential novel mechanistic insights between oxidative stress and memory biomarkers, including BNDF and CREB pathways, to modulate the pathogenesis of AD-like symptoms.

**Methods:**

Behavioral deficits were studied in terms of biochemical determination of lipid peroxidation and acetylcholinesterase activities. In addition, the study looked at the immunohistochemistry of BDNF and CREB against scopolamine-induced AD-like symptoms. Moreover, histopathological alterations were also performed against an AD-like model. Aβ_42_ proteins immunofluorescence was performed due to its known mechanism under AD. Finally, scopolamine-induced intraperitoneal mechanisms were studied in rats to establish an AD-like model.

**Results:**

The present study findings showed that administration of TRAZ considerably improved cognitive impairments as validated by NOR and display of anti-anxiety behavior, as verified by EPM. In addition, biochemical findings confirmed that TRAZ lowered oxidative stress through LPO, reduced Aβ deposition, and decreased the AChE. Furthermore, there was a notable upregulation of BDNF and CREB signaling expression, as confirmed by the IHC.

**Conclusion:**

Overall, the study findings confirmed that TRAZ could be useful in mitigating the negative effects of scopolamine-induced cognitive impairment and lowering oxidative stress by enhancing memory indicators.

## Graphical Abstract

**Figure:**
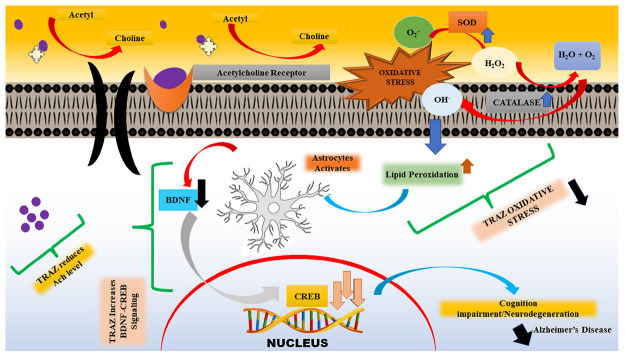
Alzheimer’s disease (AD) is a neurodegenerative condition that worsens over time and affects the brain’s cholinergic function, leading to oxidative stress in the brain, which is largely seen in AD patients. By interfering with cholinergic transmission in the central nervous system (CNS), the tropane alkaloid medication scopolamine (scop) demonstrated competitive antagonism within muscarinic acetylcholine receptors (mAChRs). The scopolamine injection causes dysregulation of the cholinergic neuronal pathway and memory circuits in the CNS. In addition, it lowers the expression of BDNF and cAMP-response element-binding (CREB) in the CNS. Therefore, BDNF and CREB may operate as innovative therapies to address memory pathological conditions and neurodegenerative illnesses like AD, the hippocampal proteins BDNF and CREB are crucial for developing long-term memory.

## 1 Introduction

Neurodegenerative diseases associated with aging have garnered significant attention on a global scale due to their increased prevalence in recent decades. Alzheimer’s disease (AD), a chronic neurodegenerative condition, is responsible for most cases of senile dementia. It comprises approximately 60%–80% of cases of senile dementia. The primary features of AD typically involve progressive cognitive decline and memory impairments. Ongoing research is focused on uncovering the causes of AD which is believed to involve complex factors including genetics, the accumulation of amyloid beta plaques, the formation of tau protein tangles, oxidative stress, dysfunction in cholinergic activity, neuroinflammation, and cellular death (Lee et al., 2023). Therefore, AD is a cognitive impairment that gradually progresses and significantly affects one’s daily activities, and ultimately leading to high mortality rates among older adults (Garre-Olmo, 2018). The prevalence of AD is steadily growing on a global scale and is projected to triple by 2050, mainly due to the progressive aging population (Prince et al., 2014; Bassani et al., 2017). Alzheimer’s dementia affects 6.7 million Americans aged 65 and older, according to estimates. If there are no medical breakthroughs to hinder, delay, or treat AD, it is projected that this number could rise to 13.8 million by 2060 (Choi et al., 2023). However, there are currently no definitive treatments or methods discovered for AD. Therefore, using antioxidants and anti-inflammatory drugs as supplementary therapies have been demonstrated to be effective in addressing AD (Abreu-Villaça et al., 2011; Zhou et al., 2016).

Cholinergic neurons within the hippocampus play a crucial role in learning and memory processes (Khakpai et al., 2012). Thus, the administration of scopolamine (scop), which acts as an antagonist to muscarinic acetylcholine (AChE) receptors, impairs short-term and spinal memory in animal models and humans. These memory deficits mimic the cognitive impairments observed in AD pathology (Klinkenberg and Blokland, 2010; Tang, 2019). Furthermore, in animal models, scop has been extensively utilized to induce learning and memory impairments by elevating the levels of Acetylcholinesterase (AChE) and lipid peroxidation (LPO) within the hippocampus (Xue et al., 2022). Additionally, due to its crucial function in synaptic plasticity, brain-derived neurotrophic factor (BDNF) is one of the prospective therapeutic targets in Alzheimer’s disease (AD). Low levels of BDNF are thought to have an impact on both the internal and external functioning of the brain by altering physiological processes in neurological disorders, including AD. It is crucial to preserve synaptic plasticity and support the hippocampus’s ability to store memories. This is done by triggering the transcription factors CREB and CREB-binding protein (CBP), which subsequently produce proteins related to brain adaptability, stress resilience, and cell viability. BDNF signaling pathways control the expression of genes. As a blood biomarker for AD patients, plasma BDNF levels estimate BDNF levels in the hippocampus (Sacks et al., 2018). In this context, recent information indicates that Aβ primarily diminishes the phosphorylation of cyclic adenosine monophosphate (cAMP) response element binding protein (CREB protein, subsequently reducing BDNF. Numerous investigations have demonstrated that Aβ inhibits CREB-mediated transcription. According to earlier research, AD-related brain damage impairs CREB-mediated gene expression. Additionally, CREB-regulated BDNF levels are declining in the AD post-mortem brain. It is possible to link cognitive loss to AD’s impaired neurotrophic factor signaling pathways. For the regulation of BDNF and the development of memories, CREB is a crucial transcriptional factor. It is important to note that during LTP, CREB-mediated gene expression is elevated in the hippocampus (Yeh et al., 2018).

Trazodone hydrochloride (TRAZ) is an antidepressant medication that belongs to the class of drugs known as serotonin antagonists and reuptake inhibitors. In recent years, there has been some interest in exploring the potential therapeutic effects of TRAZ in AD. Some studies have suggested that TRAZ may have neuroprotective properties and could modulate certain pathological processes associated with AD, such as amyloid-beta accumulation and neuroinflammation (Fatima et al., 2017).

## 2 Materials and method

### 2.1 Drugs and chemicals

All the chemicals and reagents including, BSA, Ethanol, o-phosphoric acid, Butyl hydroxytoluene, Coomassie brilliant blue G dye, and Acetylthiocholine iodide were of analytical grade and were obtained from various manufacturers. Scop (#1610001) and trazodone hydrochloride (#PHR3372) were acquired from Sigma-Aldrich (St. Louis, MO, USA).

### 2.2 Animal procurement

A total of 24 Wistar rats, with weights ranging from 200 to 250 g, were obtained with prior approval from the Institutional Animal Ethics Committee (IAEC) at Jamia Hamdard, adhering to the regulations set forth by the Committee for the Prevention and Supervision of Animal Experiments (CPCSEA). The rats were acclimated in standard laboratory conditions, residing in polypropylene cages, in a room with a temperature of 25 ± 2°C and a relative humidity of 60 ± 5%. They were given unrestricted access to a commercial pellet diet and water *ad libitum*.

### 2.3 Experimental protocol

**FIGURE 1:**
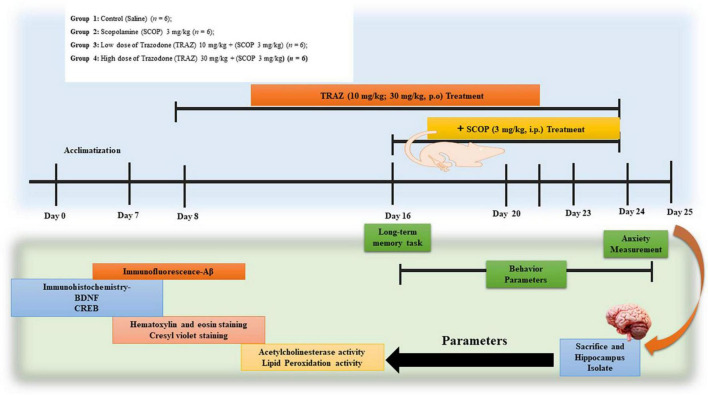
An experimental protocol was followed in this study. IAEC approved the animal testing studies, and CAHF procured male wistar rats aged (220–250 g). Animals were randomly assigned to four experimental groups: control, scop (3 mg/kg; i.p.), scop + TRAZ (10 mg/kg/p.o), scop+ TRAZ (30 mg/kg/p.o) which were then housed in regular housing. All animals were subjected to scop through *i.p.*, except the control group to establish an AD-like model. The experimental protocol was designed to study the impact of scop on cognition. On the 26th day, all experimental rats were decapitated and hippocampus was isolated for molecular work and frozen in (–80°C) for long-term use and the whole brain was fixed in (4% paraformaldehyde) for histopathological parameters.

Twenty-four rats were randomly assigned to four groups (*n* = 6), except for the control group each group was treated with scop (3 mg/kg; i.p). Group 1 (control), group 2 [scop (3 mg/kg, i.p)] only, group 3 [TRAZ (10 mg/kg) + scop 3 mg/kg)], group 4 [TRAZ (30 mg/kg) + scop (3 mg/kg)] was administered into the wistar rats (Aygun, 2020). TRAZ and scop were dissolved in nuclease-free water. The dose and route of administration were based on previous literature (Lam et al., 2016). Scopolamine injections were given intraperitoneally for 9 days and TRAZ was given orally for 18 days as depicted in Figure 1.

### 2.4 Cognitive and behavioral comorbidities

#### 2.4.1 NOR measurement

The Novel Object Recognition (NOR) test was conducted to evaluate non-spatial and short-term memory in rats, following the protocol outlined by Bevins and Besheer (2006). A rectangular box with dimensions of 40 cm × 35 cm × 40 cm served as the testing apparatus. The procedure involved three phases: habituation, familiarization and retention. Any-maze software (ANY MAZE, Video tracking Software, US) was utilized for observing animal activity and measuring time. During the habituation phase, each rat was introduced to the box environment for a period of 10 min to acclimate. Subsequently, the familiarization phase began, where two identical objects (FO1 and FO2) were positioned in opposing corners of the box. Rats were given 5 min to explore both objects. After a 1-h interval, the test phase evaluated the animal’s spontaneous behavior. In this phase, one of the familiar object was replaced with a novel object (F&N), and rats were again allowed 5 min to explore both objects (Rahman et al., 2019).

#### 2.4.2 Anxiety measurement

In the elevated plus maze (EPM) test, the animals were placed at the interaction point of the open arm. They were positioned to face the open arm and left to freely explore the maze for a period of 5 min. The duration of time spent by the animals in both the closed and open arms was meticulously observed and recorded while they actively explored the maze. Special care was taken to prevent any sudden noise disturbances that could potentially impact the behavior of the animals during the test (Bradford, 1976; Walf and Frye, 2007). The software used to perform EPM was AnyMaze (ANYMAZE, Video Tracking Software, US).

### 2.5 Preparation of brain homogenate

The brain was delicately removed from the skull and rinsed with ice-cold phosphate buffer (pH-7.4). Subsequently, the hippocampal tissues were separated from the brain and blended in ice-cold phosphate buffer (0.1 M; pH-7.4) using a centrifugal force of 10,000 *g* for 15 min at 4 °C. Following centrifugation, the resulting supernatants were accurately collected and preserved at −80 °C to maintain the biochemical constituents for subsequent analysis. To determine the protein concentration in the hippocampal samples, the Bradford method (Ellman et al., 1961) was employed. Bovine serum albumin (10 mg/ml) was used as a standard for calibration and comparison.

### 2.6 Assessment of biochemical parameters

#### 2.6.1 Acetylcholinesterase activity determination

To quantify the level of acetylcholinesterase (AChE) in the hippocampus, Ellman’s method was employed (Utley et al., 1967). A volume of 0.005 ml of the supernatant was mixed with 0.1 ml of Ellman’s reagent, also known as 5,5-dithio-bis-(2-nitrobenzoic acid) or DTNB, along with 0.1 ml of acetylthiocholine iodide, and 3 ml of sodium phosphate buffer (pH-8). The change in absorbance was measured at 412 nm using HITACHI U-2910 spectrophotometers at 30-s intervals over a duration of 2 min. The results were reported as moles of ATC hydrolyzed per minute per milligram of protein.

#### 2.6.2 Quantification of lipid peroxidation

The assessment of lipid peroxidation in the hippocampus was carried out by measuring the quantity of TBARS (Thiobarbituric Acid Reactive Substances) (Deiana et al., 2022). In 0.1 ml of tissue homogenate, 10 mM Butylated hydroxytoluene and 0.67% thiobarbituric acid (TBA) were combined, followed by centrifugation at 4000 *g* for 10 min. The test tubes containing this mixture were then subjected to incubation in a boiling water bath for 90 min. The resulting pink coloration was measured spectrophotometrically at 535 nm using Perkin Elmer Lambda 20 spectrophotometers. The obtained results were expressed in nanomoles of TBARS formed/h/g of tissue (Ashafaq et al., 2017).

### 2.7 Histopathological analysis of the brain

#### 2.7.1 H & E staining

Hematoxylin and Eosin (H&E) staining was performed for histological investigations. The brain of the rats was subjected to perfusion with 4% paraformaldehyde, followed by fixation using formalin. Post-fixation brain tissue was embedded in paraffin for a duration of 4 h. Paraffin blocks were then prepared, and coronal sections of the hippocampal CA4 and dentate gyrus regions were obtained using a microtome, with each section measuring 5 μm. These sections were mounted on silicone-coated slides and subjected to a series of treatments, including deparaffinization using xylene, rehydration through graded ethanol solutions, and staining with hematoxylin-eosin (H & E). Hippocampal neuron morphology was observed and imaged at a 20X magnification using a Zeiss microscope (Rahman et al., 2020).

#### 2.7.2 Cresyl violet staining of the hippocampus

Cresyl violet is a staining method used to visualize the Nissl substance, which is located within neurons. To perform the staining, a 0.1% Cresyl violet solution was applied to the sections. Following a 15-min incubation period for the stain to take effect, the excess dye was removed by washing the slides with 70% ethanol. The stained neurons were then observed and images were captured using a Carl Zeiss microscope at a magnification of 20X under a light microscope (Salman et al., 2022).

### 2.8 Immunohistochemical analysis of hippocampal tissue

Immunohistochemical staining was carried out following the protocol detailed in a previous study (Rahman et al., 2020) In summary, the animals were anesthetized and then perfused with cold normal saline. Subsequently, they were fixed with a 4% paraformaldehyde solution, and tissue sections were sliced from the paraffin-embedded specimen using a microtome. After removing paraffin using xylene and dehydrating with varying concentrations of graded ethanol, antigen retrieval through boiling in citrate buffer. Following the washing step, a blocking procedure was performed, and the sections were then subjected to incubation with the primary antibody targeting BDNF (1:100; lot no. #XK3756013; Invitrogen), CREB (1:100; #D76D11; CST), p CREB (1:100; #9198; CST) and KI-67 (1:100; #34330; CST) overnight at 4 °C. Afterward, following the washing step, the samples were subjected to incubation with a secondary antibody (1:1000; CST). Subsequently, staining was carried out using 3,3-Diaminibenzidine (DAB; Sigma Aldrich, USA) in butyl phthalate Polystyrene Xylene) and examined using a fluorescence microscope at a 20X magnification (Zeiss Microscopy; ZEN Blue Edition).

### 2.9 Immunofluorescence study of the hippocampus

Immunofluorescence (IF), a crucial immunological technique, is employed to detect and pinpoint various antigens within different cell samples and tissues. In the case of brain tissue slides preserved in formalin and embedded in paraffin, the following steps were carried out for immunofluorescence. First, deparaffinization and hydration procedures were executed on 3 μm thick paraffin sections. Subsequently, these sections underwent heat-induced antigen retrieval in a citrate buffer with a pH of 6.0. For hippocampal sections, Triton-X (0.3%) was applied and left to incubate for 30 min at room temperature in a blocking solution containing 1.5% (wt/vol) bovine serum albumin (BSA; Woelfle and Boeckers, 2021). The primary antibodies (Aβ, CST, and D3D2N) were diluted using phosphate buffer and then incubated overnight at 4 °C. To remove unbound antibodies, the sections were rinsed three times in 1x phosphate buffer. Subsequently, the secondary antibody (DyLight 488, goat anti-mouse IgG) was applied to the sections after being diluted 1:200 in phosphate buffer and left to incubate for an hour at room temperature. The slides were then mounted with 4’6-diamidino-2-phenylindole (DAPI), which was prepared from a stock solution of 10 mg/ml and diluted at a ratio of 1:10,000 for nuclei counterstaining (Fluoroshield; GTX30920). After a buffer wash and mounting, the slides were examined under a microscope.

### 2.10 Statistical analysis

The data obtained in our study were analyzed using Graph Pad Prism 8.0 (San Diego, CA, United States). All values were presented as the mean ± standard error of the mean (S.E.M). One-way analysis of variance (ANOVA) followed by Tukey’s post-hoc analysis; uncorrected Fisher’s LSD multiple comparison test; Newman-keuls multiple comparison test; and Tukey’s multiple comparison tests were employed to analyze the data acquired from the parameters. All values were expressed as mean ± s.e.m and the level of significance at *p* < 0.05. Statistical significance is denoted as *p < 0.05 vs. control group, #*p* < 0.05 and ##*p* < 0.01 vs. scopolamine group, and ^*p* < 0.05 for comparisons between trazodone doses.

## 3 Results

### 3.1 TRAZ mitigates scopolamine induced cognitive impairment validated by Novel Object Recognition (NOR)

**FIGURE 2:**
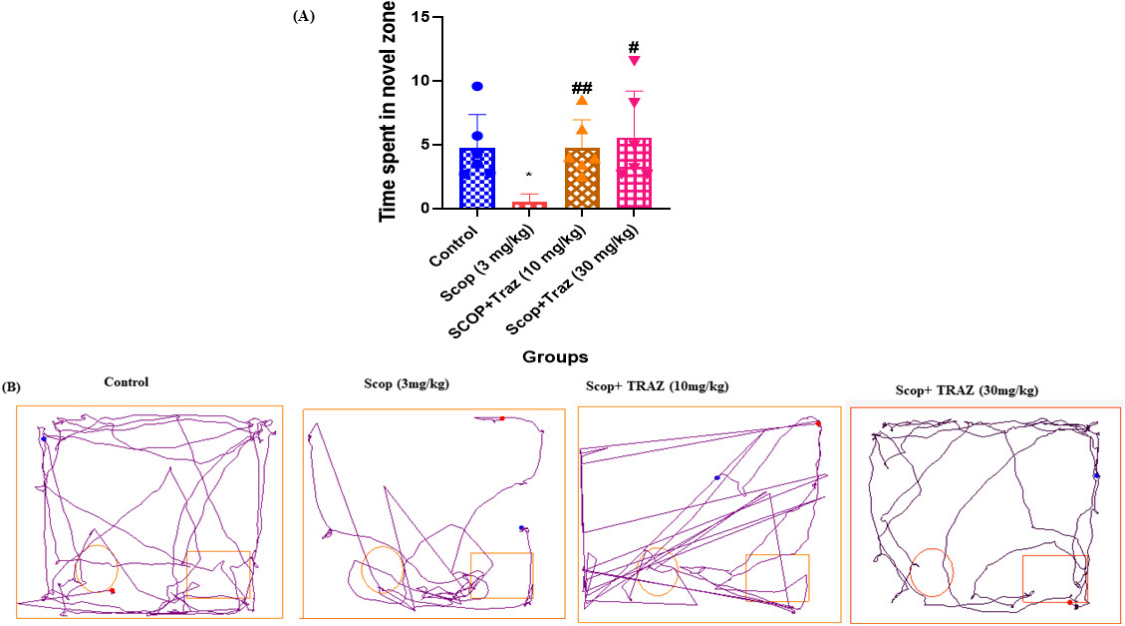
To study the effect of TRAZ on cognition **(A)** showed time spent in novel zone, which is significantly decreased in the scop-induced group (*p < 0.05) as compared to control. Traz-treated doses (10 and 30 mg/kg, respectively) significantly increased the time spent in novel zone (^##^*p* < 0.01 and ^#^*p* < 0.05) as compared to the scop-induced group. **(B)** showed a track plot generated by the software AnyMaze. Data are represented as mean mean ± s.e.m, followed by ANOVA with uncorrected Fisher’s LSD multiple comparison tests, where *n* = 6.

The time spent in the novel zone of the scop-treated group was significantly lower as compared to the control group (*p < 0.05). TRAZ (10 and 30 mg/kg, respectively) showed a significantly amount of time spent in the novel zone than scop-treated group (^##^*p* < 0.01 and ^#^*p* < 0.05) (Figure 2A). Data are represented as mean ± s.e.m, followed by ANOVA with uncorrected Fisher’s LSD tests, where *n* = 6 (Figure 2B).

### 3.2 TRAZ restores hippocampal anxiety confirmed by elevated plus maze (EPM)

**FIGURE 3:**
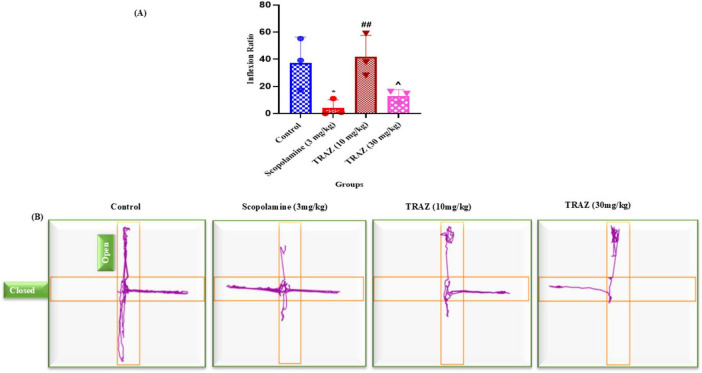
To study the effect of TRAZ on anxiety. **(A)** Showed an inflexion ratio, which is significantly decreased in the scop-induced group (*p < 0.05) as compared to control. TRAZ-treated doses showed a positive effect by increasing the inflexion ratio (^##^*P* < 0.01) as compared to the scop-induced group. TRAZ (10 mg/kg) significantly increased the inflexion ratio as compared to TRAZ (30 mg/kg) (^*P* < 0.05) **(B)** showed a track plot generated by the software AnyMaze. Data are represented as mean ± s.e.m, followed by ANOVA with uncorrected Fisher’s LSD multiple comparison tests, where *n* = 3.

The inflexion-H ratio of the scop-treated group was significantly lower than that of the control group (*p < 0.01). TRAZ (10 mg/kg and 30 mg/kg) showed a significantly higher inflexion ratio than scop (#*p* < 0.05) (Figure 3A). Data are represented as mean ± s.e.m, followed by ANOVA with uncorrected Fisher’s LSD multiple comparison tests, where *n* = 6 (Figure 3B).

### 3.3 TRAZ improved hippocampal lipid peroxidation and acetylcholinesterase (AChE) activity

**FIGURE 4:**
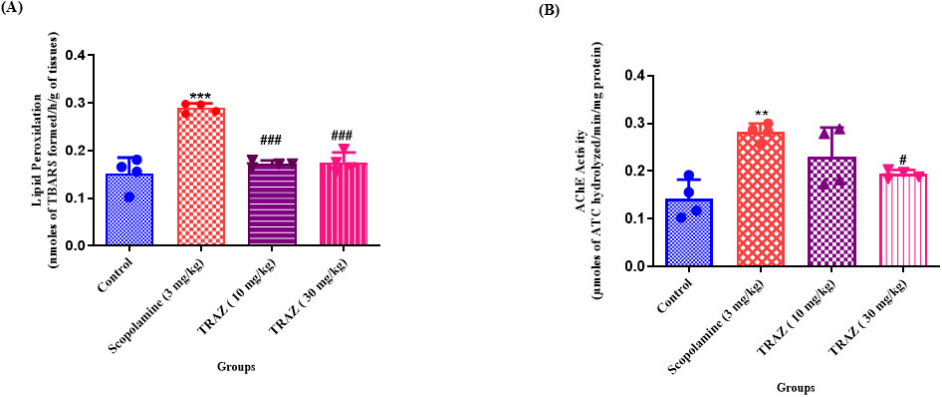
To evaluate the oxidative stress and cognitive effects of TRAZ against a scop-induced AD-like model. **(A)** Experimental results showed a significant increase in the rate of TBARS (***p < 0.001) as compared to the control. TRAZ-treated doses indicate a lower level of TBARS (^###^*P* < 0.001) as compared to the scop-induced group. **(B)** AChE level is significantly increased in the scop-induced group as compared to the control. The AChE level is significantly decreased in TRAZ (30 mg/kg) (^#^*p* < 0.05). Data are represented as mean ± s.e.m, followed by ANOVA with Tukey’s and Newman-keuls multiple comparison tests, where *n* = 4. **Symbol shows the significance between control and diseased group.

Lipid peroxidation (LPO) is widely regarded as a common and highly harmful type of oxidative stress in neurons, leading to membrane impairment and the generation of numerous secondary byproducts (Salman et al., 2022). Scop administration significantly increased the TBARS content (***p < 0.001) in comparison to the control. TRAZ-treated rats (10 mg/kg and 30 mg/kg) significantly reduced the level of TBARS (^###^*p* < 0.001) as compared to scop [F_ (3,12)__=_ 35.44] (Figure 4A). By the cholinergic hypothesis, there is a substantial increase in AChE activity in AD, resulting in the degradation of Acetylcholine (ACh). Rats given scop showed a significant (**p < 0.01) increase in AChE activity in comparison to the control group. When TRAZ was administered at a dose of 30 mg/kg, there was a notable downregulation (#*p* < 0.05) in comparison to the group that received scop injection. On the other hand, TRAZ did not significantly lower AChE activity at 10 mg/kg [F _(3,12)_ = 9.599]. (Figure 4B). Data are represented as mean ± s.e.m. One ANOVA with Turkey’s multiple comparison tests, where *n* = 4.

### 3.4 TRAZ upregulated the expression of brain-derived neurotrophic factor (BDNF)

**FIGURE 5:**
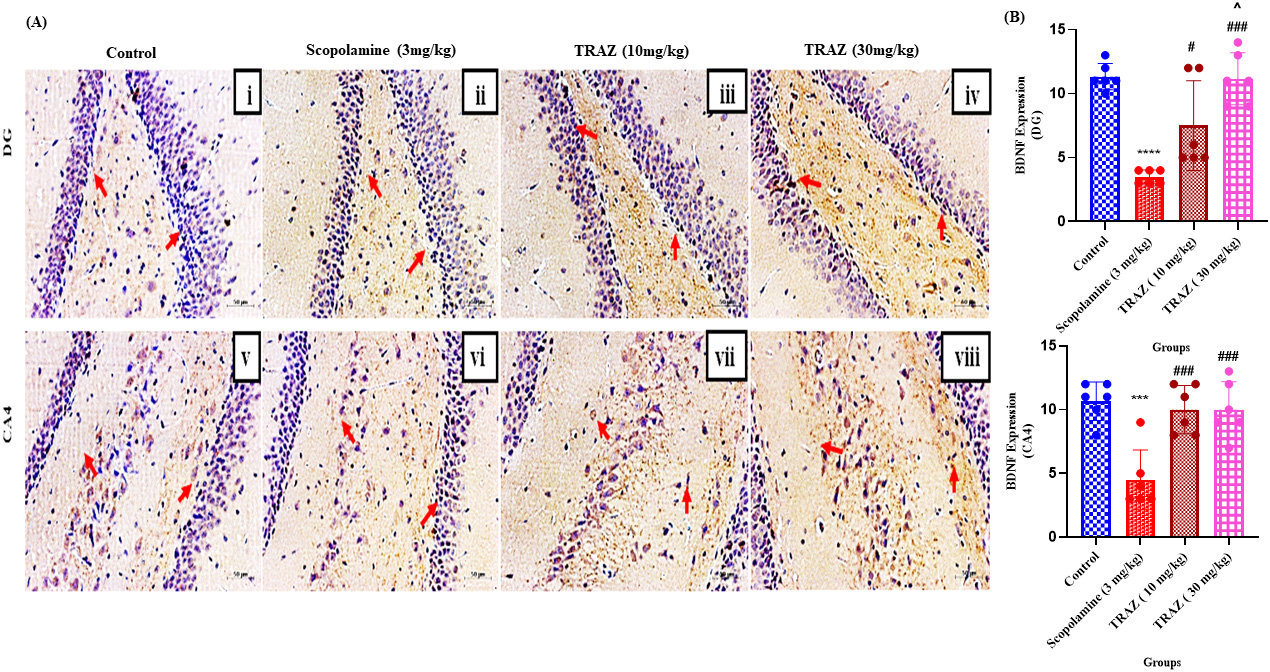
BDNF expression is increased by TRAZ administration against scop-induction in the sub-hippocampal region. **(A)** The arrow indicated the BDNF protein expression in the sub-regions of the hippocampus; CA4 and DG regions of the rat brain tissue. **(B)** In both the regions of the hippocampus, DG and CA4, the expression of BDNF was significantly lower expressed in the scop-induced group (^#^*p* < 0.05;^ ###^*p* < 0.001) as compared to the scop-induced group. TRAZ (30 mg/kg) significantly showed higher expression in comparison to TRAZ lower dose (^*p* < 0.05). In the CA4 region, BDNF expression is significantly increased in TRAZ-treated dosed (^###^*p* < 0.001) compared to the scop group in panel **(B)**. Data was displayed as mean ± SD, followed by ANOVA with Turkey’s multiple comparison test, where *n* = 6. ***, ****Symbol shows the significance between control and diseased group.

The scop-induced group exhibited significantly reduced expression of BDNF in both the DG and CA4 areas of the hippocampal regions (****p < 0.001; ***p < 0.001) when compared to the control group. Comparing the TRAZ-treated group to the scop-induced group, the BDNF expression in the DG region was considerably higher (#*p* < 0.05; ###*p* < 0.001). When compared to TRAZ at a lower dose, TRAZ (30 mg/kg) substantially showed greater expression (^*p* < 0.05). TRAZ-treated dosages dramatically boost BDNF expression in the CA4 region (###*p* < 0.001) as compared to the scop group (Figure 5B). For the DG and CA4 regions, the *F*-values are [F(3,2) = 18.43 F(3,20) = 12.30]. Data was displayed as mean ± SD, followed by ANOVA with Tukey’s multiple comparison test, where *n* = 6 (Figure 5A).

### 3.5 TRAZ improves the expression of (cAMP) response element binding protein (CREB) protein

**FIGURE 6:**
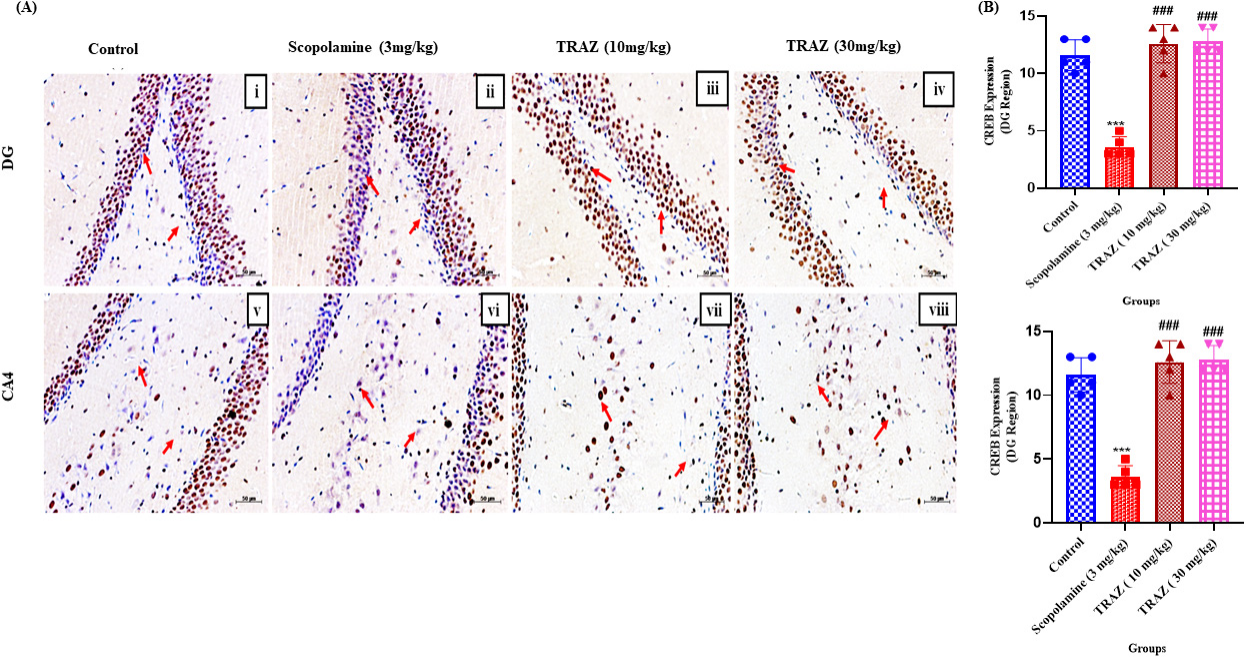
CREB expression is increased by TRAZ administration against scop-induction in the sub-hippocampal region. **(A)** The arrow indicated the CREB protein expression in the sub-regions of the hippocampus; CA4 and DG regions of the rat brain tissue **(B)** The expression of CREB was significantly decreased in the scop group (***p < 0.001) in the DG region and (*p < 0.05) in the CA4 region compared to the control group. The significantly higher CREB in the TRAZ group (^###^*p* < 0.01) in the DG region and (^##^*p* < 0.01) in the CA4 region compared to the scop group in panel **(B)**. Data was presented as mean ± SD, followed by ANOVA with Tukey’s multiple comparison test, where *n* = 5.

In the DG region and the CA4 region, the scop group exhibited a substantial decrease in CREB expression (***p < 0.001) compared to the control group. In comparison to the scop group, the TRAZ group exhibited significantly increased expression of CREB (^###^*p* < 0.001) in the DG and CA4 regions (Figure 6B). The *F*-value for DG and CA4 region is [F_(3,1)__=_ 58.62; F_(3,16)__=_ 9.632]. Data was presented as mean ± SD, followed by ANOVA with Tukey’s multiple comparison test, where *n* = 5 (Figure 6A).

#### 3.5.1 TRAZ improves the expression of phosphorylated (cAMP) response element binding protein (p CREB) protein

**FIGURE 7:**
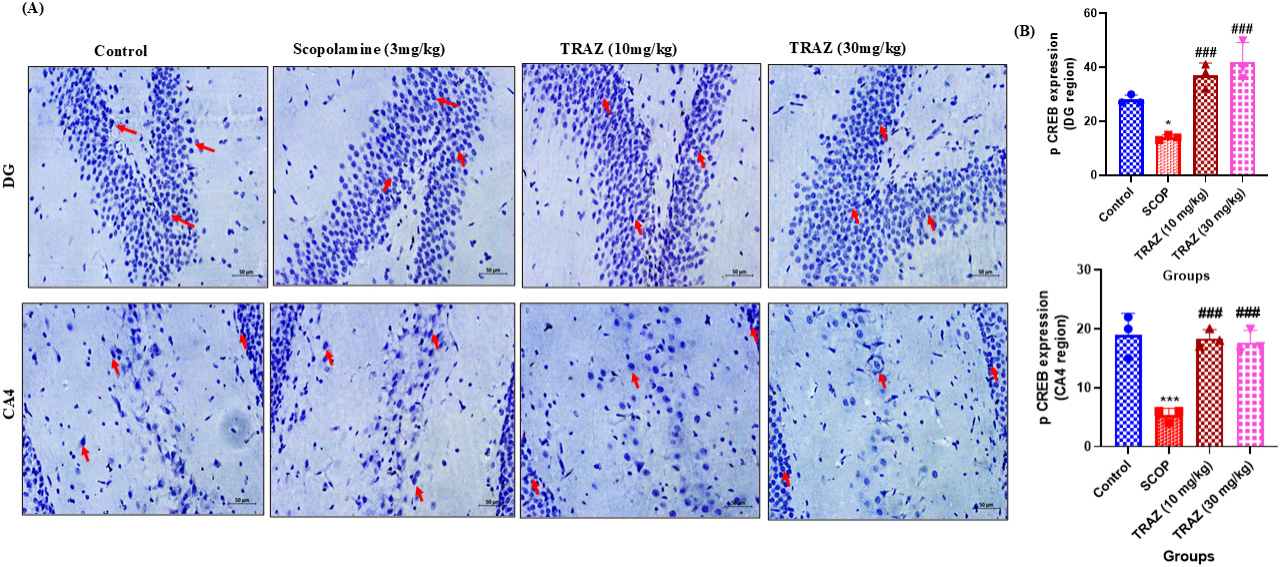
p-CREB expression is increased by TRAZ administration against scop- induction in the sub-hippocampal region. **(A)** Representative immunohistochemical images showing p-CREB expression (indicated by arrows) in the CA4 and DG regions of the hippocampus across experimental groups. **(B)** The expression of p-CREB was significantly decreased in the scop group (*p < 0.05) in the DG region and (***p < 0.001) in the CA4 region compared to the control group. The significantly higher p-CREB in the TRAZ group (^###^*p* < 0.001) in the DG region and (^###^*p* < 0.001) in the CA4 region compared to the scop group in panel **(B)**. Data was presented as mean ± SD, followed by ANOVA with Tukey’s multiple comparison test, where *n* = 3.

In the DG region and the CA4 region, the scop group exhibited a substantial decrease in p- CREB expression (*p < 0.05), (***p < 0.001), respectively, compared to the control group. In comparison to the scop group, the TRAZ group exhibited significantly increased expression of p-CREB (^###^*p* < 0.001) in the DG and CA4 regions (Figure 7B). The *F*-value for DG and CA4 region is [F_(3, 8)_ = 23.52; F_(3, 8)_ = 24.31]. Data was presented as mean ± SD, followed by ANOVA with Tukey’s multiple comparison test, where *n* = 3 (Figure 7A).

### 3.6 TRAZ improves the expression of Aβ in the hippocampal section

**FIGURE 8:**
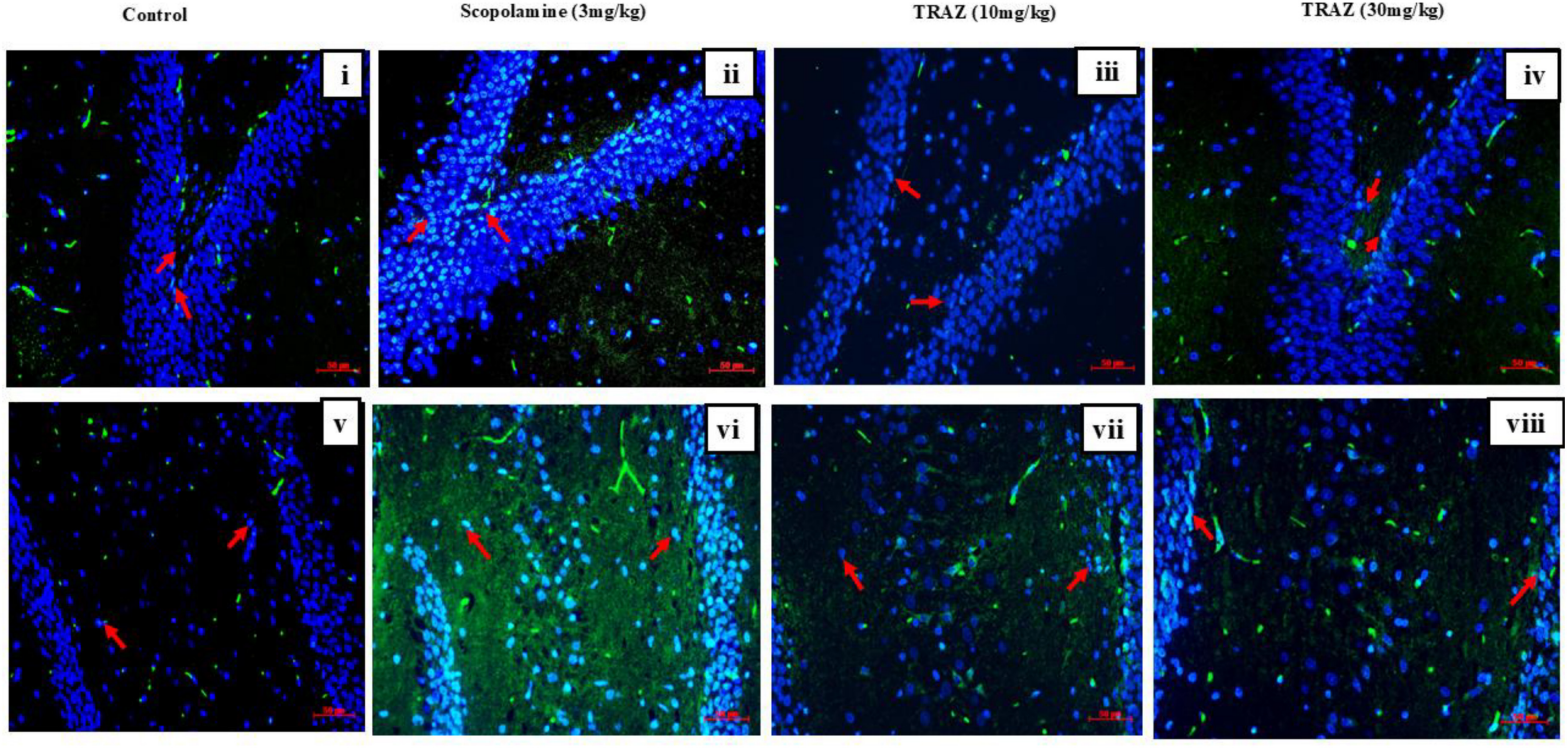
TRAZ improves the expression of Aβ in the hippocampal section: Qualitative analysis of Aβ expression in the sub-regions of the hippocampus indicated by the arrows. Immunofluorescence staining showed increased expression of Aβ level (cyan) in the sub-hippocampal regions; DG and CA4 of scop-induced AD-like model group as compared to control. Treatment with TRAZ lowered the expression of Aβ as compared to the diseased group. DAPI (blue) was used to stain nuclei. Images were captured with a Zeiss microscope at 20x.

Alzheimer’s disease is the multifactorial and the most conspicuous disorder of neuron disease. AD pathogenesis is closely associated with Aβ aggregation. In the present study, we estimated the qualitative analysis of Aβ expression in the sub-regions of the hippocampus indicated by the arrows. Immunofluorescence staining showed increased expression of Aβ level (cyan) in the sub-hippocampal regions (DG and CA4) to the scop-induced AD-like model group as compared to the control. Treatment with TRAZ lowered the expression of Aβ as compared to the scop group (Figure 8). DAPI (blue) was used to stain nuclei. Images were captured with a Zeiss microscope at 20x.

### 3.7 Hematoxylin and eosin staining confirms the restoration of damaged tissue by TRAZ treatment

**FIGURE 9:**
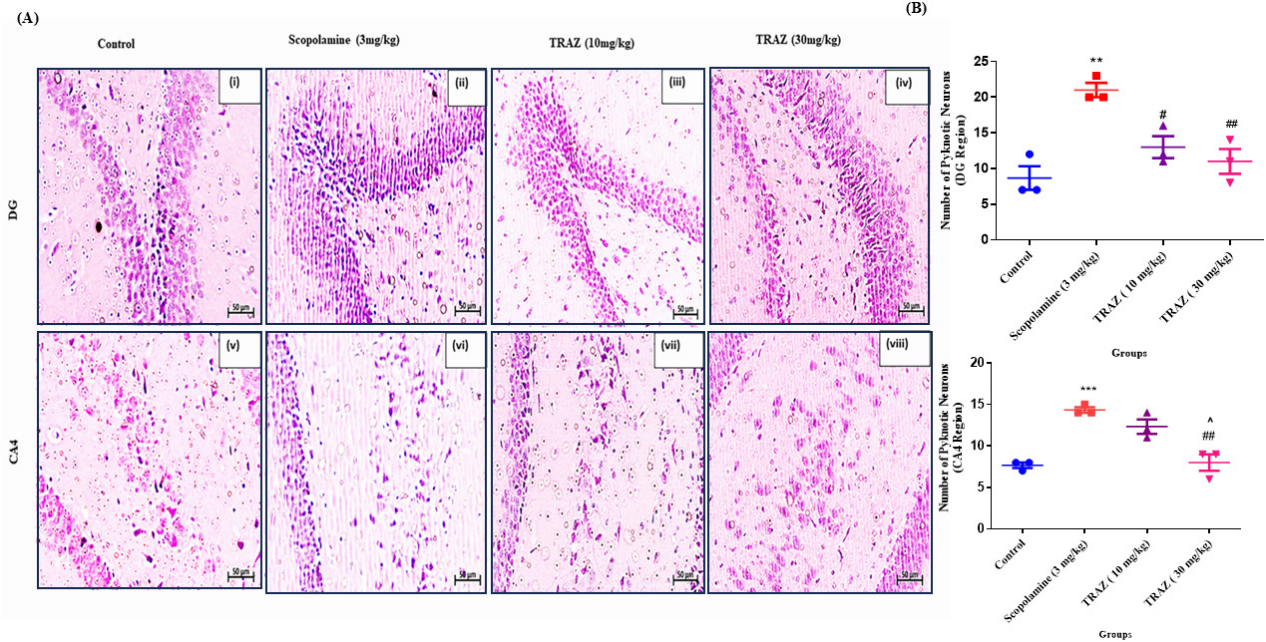
H&E staining shows the reduction of pyknotic neurons in the hippocampus region by TRAZ administration **(A)** Qualitative analysis of pyknotic neurons in the sub-regions of the hippocampus indicated by the arrows. **(B)** The number of pyknotic neurons in the scop-induced group was significantly increased (**p < 0.01) as compared to the control. TRAZ (10 and 30 mg/kg) restored the damaged neurons significantly (^#^*p* < 0.05; ^##^*p* < 0.01) in comparison to the scop-induced group. In the CA4 region of the brain hippocampus, the pyknotic nuclei are significantly reduced in TRAZ (30 mg/kg) as compared to TRAZ (10 mg/kg), (^*p* < 0.05). Here, data are represented as mean ± s.e.m and one-way ANOVA repeated measures with Turkey’s multiple comparison tests where *n* = 9, Magnification 20x. ***Symbol shows the significance between control and diseased group.

To identify the histological alterations, we performed the hematoxylin and eosin staining in the sub-hippocampal region and quantified the pyknotic neurons in the hippocampal region after scop administration. The arrows indicate the pyknotic neurons in the sub-regions of the hippocampus; CA4, and DG region in rat brain tissue (Figure 9A). The number of pyknotic neurons in sub-hippocampal regions in the scop group was significantly increased (**p < 0.01; ***p < 0.001) as compared to the control group, respectively. The TRAZ-treated group showed a significant reduction in pyknotic nuclei (^#^*p* < 0.05; ^##^*p* < 0.01) as compared to the scop group in the DG and CA4 region [F_(3,8)__=_ 12.60]. The pyknotic nuclei are significantly reduced in TRAZ (30 mg/kg) as compared to TRAZ (10 mg/kg) (^*p* < 0.05) in a CA4 region [F_(3,8)__=_ 21.54] (Figure 9B). Here, data is represented as mean ± s.e.m and one-way ANOVA repeated measures with Tukey’s multiple comparisons tests where *n* = 3,4 Magnification 20X.

### 3.8 TRAZ recovers the neuronal loss confirmed by cresyl violet staining

**FIGURE 10:**
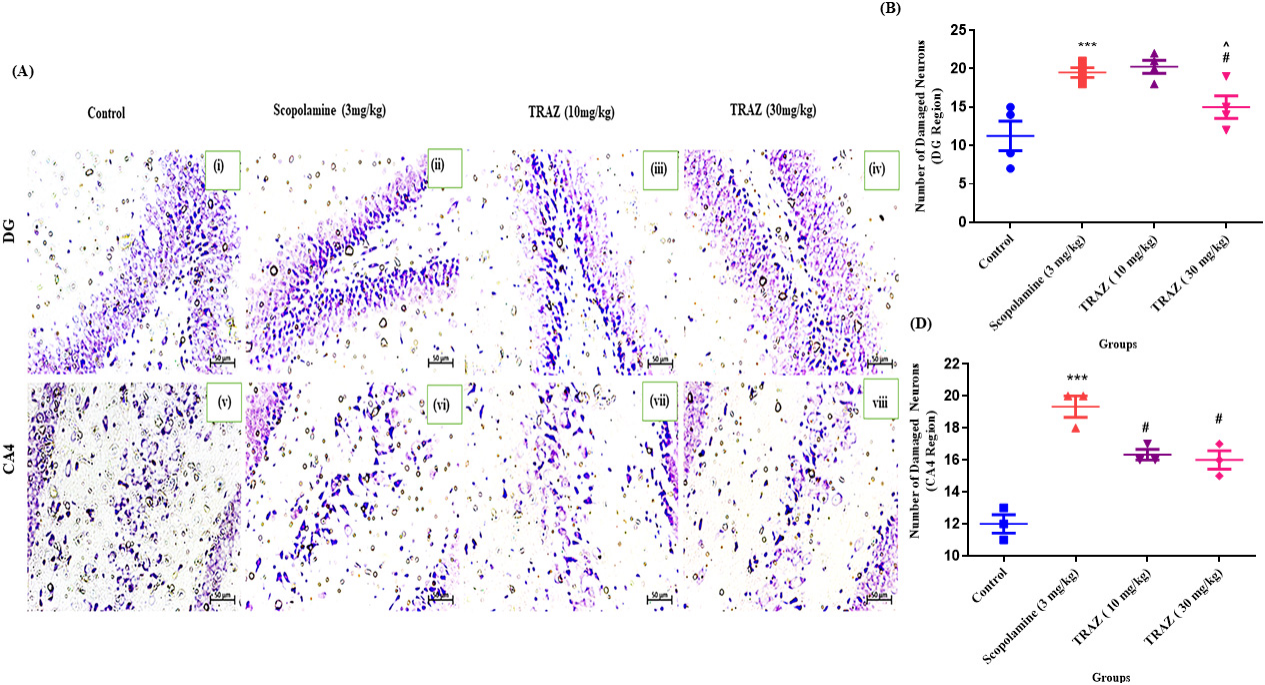
CV staining confirms the reduction of damaged neurons in the hippocampus region by TRAZ administration **(A)** Qualitative analysis of damaged neurons in the hippocampal regions indicated by the arrows. **(B)** The number of damaged neurons in the hippocampus region was significantly increased (***p < 0.001) and (***p < 0.001) in DG and CA4 region, respectively as compared to control. TRAZ (10 mg and 30 mg) restored the damaged neurons significantly (^#^*p* < 0.05) in comparison to the scop-induced AD-like group. The TRAZ (30 mg/kg) is significantly increased (^*p* < 0.05) in comparison to TRAZ (10 mg/kg). Here, data are represented as mean ± s.e.m and one-way ANOVA repeated measures with Tukey’s multiple comparison tests where *n* = 7, Magnification 20x.

To identify the histological alterations, we performed the CV staining in the sub-hippocampal region and quantified the damaged neurons in the hippocampal region after scop administration. In rat brain tissue, the arrows represent the morphology of neurons in the CA4 and DG areas (two sub-regions of the hippocampus) (Figure 10A). The number of damaged neurons in the sub-hippocampal DG and CA4 regions was significantly increased (***p < 0.001) as compared to the control. TRAZ-treated group of dose (30 mg/kg) significantly reduced the neuronal damage (^#^*p* < 0.05) as compared to scop in both DG and CA4 regions. When compared to TRAZ (10 mg/kg), TRAZ (30 mg/kg) considerably lowers the number of damaged neurons in the DG area (^*p* < 0.05) (Figure 10B). Data are represented as mean ± s.e.m, and statistical analysis by one-way ANOVA repeated measures with Tukey’s multiple comparisons [F_(3,8)__=_ 29.67] and Uncorrected Fisher’s LSD tests [F_(3,12)__=_ 10.01] where *n* = 3,4 Magnification 20X.

### 3.9 TRAZ promotes neuronal proliferation as indicated by Ki-67 expression

**FIGURE 11:**
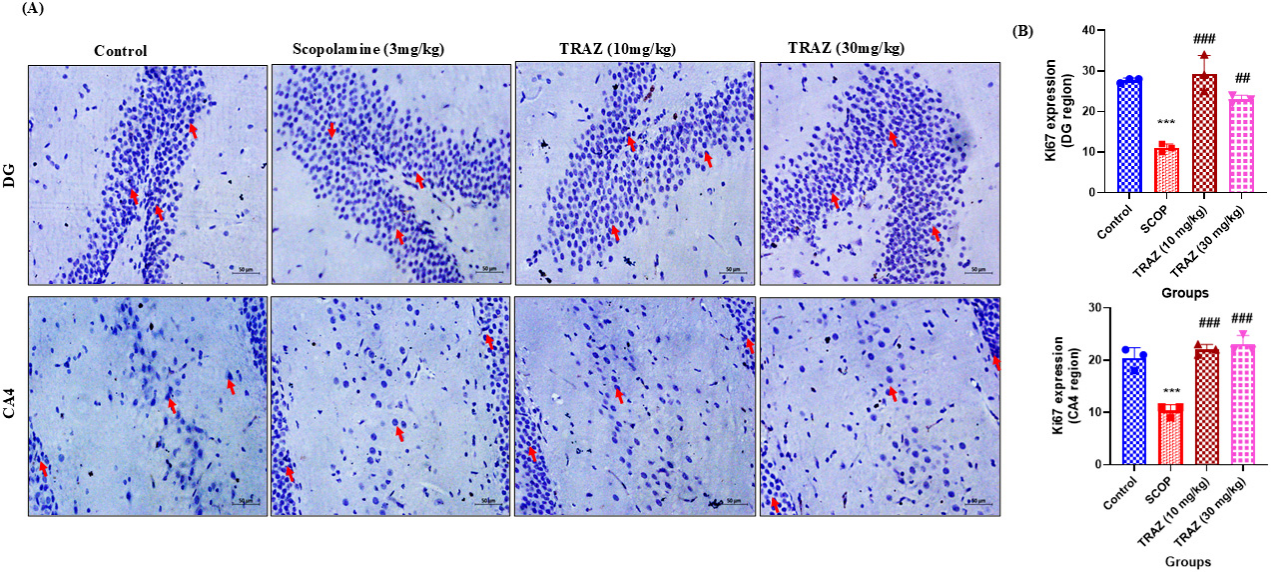
TRAZ administration increases Ki-67 expression against scop-induction in the sub-hippocampal region **(A)** Representative immunohistochemical images showing Ki-67–positive cells (indicated by arrows) in the dentate gyrus (DG) and CA4 regions of the hippocampus across experimental groups. **(B)** The expression of Ki-67 was significantly decreased in the scop group (***p < 0.001) in the DG region and (***p < 0.001) in the CA4 region compared to the control group. The significantly higher Ki-67 in the TRAZ (10 mg/kg) group (^###^*p* < 0.001) and (^##^*p* < 0.01) in the TRAZ (30 mg/kg) in the DG region and (^###^*p* < 0.001) in the CA4 region compared to the scop group in panel **(B)**. Data was presented as mean ± SD, followed by ANOVA with Tukey’s multiple comparison test, where *n* = 3.

In the DG region and the CA4 region, the scop group exhibited a substantial decrease in p Ki-67 expression (***p < 0.001) compared to the control group. In comparison to the scop group, the TRAZ (10 mg/kg) group exhibited significantly increased expression of Ki-67 (^###^*p* < 0.001) and the TRAZ (30 mg/kg) group exhibited significant increase in the expression of Ki-67 (^##^*p* < 0.01) in the DG region and in CA4 region both the TRAZ groups exhibited significant increased in the expression of Ki-67 (^###^*p* < 0.001) (Figure 11B). The *F*-value for DG and CA4 region is [F_(3, 8)_ = 36.29; F_(3, 8)_ = 42.15]. Data was presented as mean ± SD, followed by ANOVA with Tukey’s multiple comparison test, where *n* = 3 (Figure 11A).

## 4 Discussion

Alzheimer’s disease (AD) is characterized by neuronal degeneration, a decline in memory, and abnormalities in neurobehavior. It is still difficult to successfully manage the severity of the disease, even with the availability of several drugs and treatments. As a result, there is a need to develop more alternative therapies that can be used to treat AD. The function of TRAZ has been closely linked to AD after more than ten years of research. The purpose of this study was to ascertain whether or not trazodone hydrochloride affects cognition and anxiety by controlling the BDNF-CREB mechanism. An induced male Wistar rat model with scop was utilized to validate the concept.

In the current study, the NOR and EPM test was applied as a neurobehavioral model to analyze the cognitive impairment and anxiety levels in the rats, respectively (Ashafaq et al., 2017). Our study results indicated that TRAZ exhibited a notable improvement in learning and memory consolidation in the treated group compared to rats treated with SCOP. Rats treated with SCOP exhibited a marked inability to discriminate between familiar and novel objects, reflecting a clear deficit in recognition memory. Administration of TRAZ significantly alleviated this impairment, with treated animals demonstrating improved inflexion ratios compared to the control group. These improvements highlight the potential of TRAZ in restoring cognitive performance disrupted by SCOP. Interestingly, the behavioral benefits of TRAZ were observed across all treatment groups, indicating that its efficacy in enhancing memory functions did not vary with dosage. This phenomenon can be clarified by the notion that the drug reaches its maximum effect at a higher dose, and increasing the dosage beyond that point does not enhance its efficacy. While both the 10 mg/kg and 30 mg/kg trazodone doses were effective in ameliorating scopolamine-induced memory deficits in the NOR task, the EPM results showed that the 10 mg/kg dose produced a substantial anxiolytic effect, with no additional benefit observed at 30 mg/kg. This suggests a possible ceiling effect for trazodone’s behavioral actions, beyond which higher doses do not yield greater efficacy. Such non-linear or saturating dose–response relationships are typical for neuroactive drugs and may arise from differential sensitivity or receptor engagement in cognitive versus anxiety-related neural circuits. Similar patterns have been reported for other serotonergic agents in rodent models of behavior (Buchborn et al., 2018). Thus, the 10 mg/kg dose appears optimal for both cognitive and anxiolytic effects in this paradigm, and further increases may not enhance the therapeutic benefit. Therefore, these findings suggest that TRAZ had a positive effect in the scop-induced AD-like model to overcome anxiety.

In addition, we examined the role of TRAZ in the cholinergic pathway and in defense against oxidative stress. The brain is particularly susceptible to oxidative stress due to its high oxygen consumption, abundant lipid content, and relatively low antioxidant levels compared to other organs (Bridges, 1980). Additionally, it is widely recognized that the brain’s hippocampal region plays a vital role in learning, memory, and the creation of spatial memories (Heo et al., 2014). The AD model induced by scop revealed notable oxidative stress and memory impairments in a rodent model that closely resembles those observed in AD patients (Serrano and Klann, 2004). The higher TBARs in the scop-treated group compared to the control group indicated that scop injection-induced oxidative stress and altered cholinergic pathway in the hippocampus were responsible for these effects. The AChE expression level and TBARS body were reduced in TRAZ-treated groups.

Next, we examined the levels of BDNF (Brain-derived Neurotropic factor) and CREB (cAMP response element-binding protein) expression in hippocampal tissue subjected to scop-induced effects to explore TRAZ’s role in neurogenesis and synaptic plasticity. Our findings revealed a significant decrease in BDNF and CREB within the hippocampus following the scop injection. However, treatment with TRAZ led to a notable increase in the levels of both BDNF and CREB. A high dose of TRAZ showed maximum protection by increasing the levels of BDNF and showed similar effects for CREB. Apart from its previously mentioned functions, cAMP response element-binding protein (CREB) is vital in facilitating neuronal growth, promoting cellular proliferation, driving differentiation, and ensuring neuronal survival (Choi et al., 2012). Recent research has indicated that reduction in levels might be linked to the development of AD (Gómez-Palacio-Schjetnan and Escobar, 2013). BDNF safeguards neurons from various forms of brain damage and plays a crucial part in the growth and upkeep of both central and peripheral neurons (Givalois et al., 2004). In individuals with AD, it has been noted that both the precursor form and mature BDNF experience a decline in the hippocampus, even during the pre-clinical stages of the disease (Peng et al., 2005). In alignment with our findings, it is evident that the administration of TRAZ can notably increase BDNF levels in rats. These results lend support to the idea that BDNF plays a protective role in combating AD-like symptoms. Furthermore, scop-induced Aβ expression was higher than the control group. TRAZ treatment showed that enhancing BDNF-CREB signaling can reduce Aβ production or enhance the clearance (Figure 10). The observed reduction in Aβ expression following trazodone treatment may be attributed to multiple converging mechanisms. Trazodone has been reported to inhibit the PERK–eIF2α signaling pathway, which is overactivated in Alzheimer’s disease and contributes to abnormal amyloid precursor protein (APP) translation and Aβ accumulation (Halliday et al., 2015); (Rohe et al., 2009). By attenuating this stress response, trazodone may help restore normal protein synthesis and reduce Aβ burden. Moreover, activation of the BDNF–TrkB–CREB signaling axis, as observed in our study, is known to enhance neuronal resilience and promote non-amyloidogenic APP processing via upregulation of α-secretase activity (Rohe et al., 2009). Together, these mechanisms suggest that trazodone exerts a dual protective effect by reducing Aβ formation and enhancing its clearance through neurotrophic and translational control pathways.

In addition to total CREB levels, we also examined phosphorylated CREB (p-CREB), which represents the transcriptionally active form of the protein. Our findings demonstrated that scopolamine significantly reduced p-CREB expression in the DG and CA4 regions of the hippocampus, whereas trazodone treatment markedly restored p-CREB levels. Importantly, since CREB phosphorylation is primarily mediated via the BDNF–TrkB signaling pathway, the observed increase in p-CREB strongly suggests that trazodone treatment activated upstream TrkB receptors. This provides more direct mechanistic evidence that trazodone promotes neuroplasticity through BDNF–TrkB–CREB signaling, beyond simply increasing total CREB expression.

We further evaluated the effect of trazodone on neuronal proliferation using Ki-67, a nuclear marker of actively dividing cells. Scopolamine treatment markedly reduced Ki-67–positive cells in the dentate gyrus, consistent with impaired hippocampal neurogenesis. Trazodone administration significantly restored Ki-67 expression, suggesting that trazodone not only protects existing neurons but also promotes the generation of new ones. These findings indicate that activation of the BDNF–TrkB–CREB pathway by trazodone may extend downstream to enhance hippocampal neurogenesis, which is critically impaired in Alzheimer’s disease. Together, this supports a dual role of trazodone in both neuroprotection and neuro-regeneration.

It is worth noting that trazodone is most commonly prescribed in the clinic as a hypnotic for insomnia, raising the possibility that sedative effects could contribute to the behavioral differences observed. However, several aspects of our findings argue against sedation as the main explanation. In both NOR and EPM, trazodone-treated animals demonstrated increased exploration and improved performance, which would be unlikely if the drug had primarily reduced arousal. Instead, our molecular analyses showing upregulation of BDNF and CREB suggest that the improvements are more consistent with serotonergic modulation of neuroplasticity. While we cannot exclude some contribution of sleep regulation to trazodone’s overall neuroprotective profile, our data support the interpretation that serotonergic mechanisms play a central role in mediating the observed effects.

In summary, AD is known for its progressive and significant increase in neurodegeneration, which leads to brain atrophy and significant reductions in memory and cognitive function (Givalois et al., 2004). The current study found that using scop resulted in a significant amount of hippocampal neuronal damage, as demonstrated by histological staining with the use of H&E and cresyl violet. However, it showed a protective effect against this neuronal damage when administered with TRAZ (probably a therapeutic intervention). Consequently, TRAZ might present a viable treatment strategy for people suffering from neurodegenerative illnesses.

## Data Availability

The original contributions presented in this study are included in this article/supplementary material, further inquiries can be directed to the corresponding authors.
